# Divergent roles of autistic and alexithymic traits in utilitarian moral judgments in adults with autism

**DOI:** 10.1038/srep23637

**Published:** 2016-03-29

**Authors:** Indrajeet Patil, Jens Melsbach, Kristina Hennig-Fast, Giorgia Silani

**Affiliations:** 1 Scuola Internazionale Superiore di Studi Avanzati, Neuroscience Sector, Trieste, Italy; 2 Department of Applied Psychology: Health, Development, Enhancement and Intervention, University of Vienna, Austria

## Abstract

This study investigated hypothetical moral choices in adults with high-functioning autism and the role of empathy and alexithymia in such choices. We used a highly emotionally salient moral dilemma task to investigate autistics’ hypothetical moral evaluations about personally carrying out harmful utilitarian behaviours aimed at maximizing welfare. Results showed that they exhibited a normal pattern of moral judgments despite the deficits in social cognition and emotional processing. Further analyses revealed that this was due to mutually conflicting biases associated with autistic and alexithymic traits after accounting for shared variance: (*a*) autistic traits were associated with *reduced* utilitarian bias due to elevated personal distress of demanding social situations, while (*b*) alexithymic traits were associated with *increased* utilitarian bias on account of reduced empathic concern for the victim. Additionally, autistics relied on their non-verbal reasoning skills to rigidly abide by harm-norms. Thus, utilitarian moral judgments in autism were spared due to opposite influences of autistic and alexithymic traits and compensatory intellectual strategies. These findings demonstrate the importance of empathy and alexithymia in autistic moral cognition and have methodological implications for studying moral judgments in several other clinical populations.

“[Autistic people are] cold, calculating killing machines with no regard for human life!”-Facebook post by “Families Against Autistic Shooters” in response to the mass-shooting incident at Umpqua Community College, Oregon (as reported in *The New York Times* Op-Ed article “The Myth of the ‘Autistic Shooter’” by Andrew Solomon, October 12, 2015). Harmful behaviours are inherently dyadic, comprising of an agent who harms and a victim who gets harmed^1^. Accordingly, moral evaluations in healthy individuals about such behaviours hinges on two different routes to the understanding of other minds^2^: a cognitive route that represents agent’s beliefs and goals (called theory of mind (ToM) or *sociocognitive* route), while an affective route that identifies feeling states in the victim and elicits isomorphic feeling states (e.g., pain) in the observer (called empathy or *socioaffective* route).

Autism spectrum disorder (ASD) is characterized by problems with reciprocal social interaction, impaired communication, repetitive behaviours/narrow interests and impairments in the very aspects of social cognition and emotional processing necessary for proper moral reasoning^3^. Although past work has investigated impact of ToM deficits on moral judgments, the effect of empathy deficits remains to be thoroughly investigated. Furthermore, recent body of work shows that only ToM deficits are inherent to the autistic phenotype and the empathy deficits are due to co-occurring alexithymia^3^, a subclinical condition characterized by difficulty in identifying and describing subjective feeling states, difficulty in differentiating feelings from bodily sensations, and diminished affect-related fantasy^4^,^5^. Thus, the role of alexithymia in moral evaluations in autism is to date largely unexplored^6^. The current study explores these issues further.

## Moral cognition in autism: an overview

A number of prior studies have utilized variety of moral cognition tasks to explore if the capacity to judge third-party harmful behaviours is intact in ASD in the light of the deficits in social cognition and emotional functioning. This research shows that the distinction between intentional moral transgressions (that involve a suffering victim whose personal rights are violated; e.g. hitting others) and conventional transgressions (characterized by infraction of normative prohibitions but with no consequence for others’ welfare; e.g. talking out of turn) is substantially intact in children and adults with ASD^7^,^8^,^9^,^10^. These studies underscore that ASD population (both children and adults) can distinguish between *intentional* good and bad actions and have preserved moral knowledge^11^,^12^.

Although autistics do not seem to be impaired in evaluating intentional third-party harm-doings, they exhibit enduring deficits on more complex intent-based moral judgment tasks that require integration of information about mental states of the agents with the information about outcomes of these acts. In particular, they judge accidental harms more harshly, arguably due to their inability to form a robust representation of agent’s benign intentions due to ToM deficits^13^ that can be weighted up against a strong negative emotional response stemming from the victim suffering^14^,^15^,^16^,^17^,^18^ (but see Baez *et al.*^19^). Thus, this work is consistent with the profile of ASD^20^ featuring preserved psychophysiological/emotional response to others’ affective states (affective empathy) but reduced cognitive understanding about others’ internal states (ToM). This work also demonstrates how these ToM deficits modulate their moral judgments about third-party moral violations, but only when these processes need to operate in tandem with other processes (e.g., harm assessment) that provide conflicting contextual information that needs to be integrated for a final moral judgment^21^,^22^.

Despite an abundance of work focusing on role of ToM deficits on performance on intent-based judgment tasks that involve conflict between intent and consequences, there is a paucity of literature exploring how empathy deficits in ASD translate into behavioural choices in hypothetical scenarios.

### Empathy and moral condemnation of harmful behaviour

Emotions play a pivotal role in condemnation of harmful behaviours^23^ and empathy is a social emotion that plays a crucial role in such moral evaluations^24^,^25^. This is because (real or hypothetical) harmful encounters include a suffering victim and empathy allows moral judges to understand their suffering and use the resulting “gut-feelings” to either approve or disapprove of such moral actions^25^. But empathy is a multidimensional construct^26^ consisting of a cognitive component that is involved in merely understanding the emotional states in others, while affective empathy enables observers to share these feeling states in an isomorphic manner. Accordingly, affective empathy has been found to be more consequential in motivating behaviour (for a review, see Ugazio *et al.*^25^). But affective empathy itself has two disparate facets that are associated with different motivational tendencies^24^,^25^: (*i*) other-oriented *empathic concern* involves intuitions about protecting physical integrity of others and being apprehensive of any actions that result in harm to others and is associated with *appetitive* motivation to prevent harm to others; (*ii*) self-oriented *personal distress* reflects aversive feeling contingent on vicarious sharing of the others’ emotional and physical distress and a sense of loss of control in emotionally-charged harmful situations and is associated with *avoidance* motivation to escape such distressful situation.

Given this crucial role of empathy in moral condemnation of harmful behaviour, ASD would be expected to have impairments in moral judgments in situations that harness these processes. But this simplistic picture is further complicated in light of the new insights provided by the alexithymia hypothesis^3^ which postulates that only the deficits observed in the *sociocognitive* domain are unique to the autism phenotype, while the deficits associated with *socioaffective* domain are due to the co-occurring alexithymic phenotype and is not a feature of autism *per se*^27^. Although the preponderance rate of clinical levels of alexithymia in healthy population is at 10%, it is unusually prevalent (40–65%) in adults and children with ASD^28^,^29^,^30^,^31^,^32^. Therefore, it is important to account for its effects in emotional processing deficits observed in ASD, especially because trait alexithymia itself has been associated with impaired emotional processing (e.g., empathy^33^,^34^,^35^, emotion regulation^36^, emotional interoception^37^, etc.). Thus, it is likely that, when observed, the emotional processing deficits in ASD are due to the presence of elevated levels of alexithymia. Indeed, after accounting for co-occurring alexithymia, autism is no longer associated with aberrant neural activation while empathizing with others’ pain^38^, self-reported deficits on dispositional empathy^39^, or deficits in interocepting on one’s own emotional states^37^.

Thus, any investigation gauging effects of aberrant emotional skills on moral cognition in ASD should also account for effects of prevalent alexithymia. Indeed a number of recent studies have begun to explore role of alexithymia in moral judgments in both clinical^40^ and non-clinical populations^41^,^42^,^43^,^44^, but only one study thus far has investigated this issue^6^ in the ASD population and found limited support for the alexithymia hypothesis. In particular, this study^6^ revealed that moral judgments were predicted by alexithymia in healthy controls but not in individuals with ASD, which suggested that decision-making in ASD was less subject to emotional biases as they did not base their moral judgments on emotional information.

In the current study, we further investigate role of emotional processing deficits and alexithymia in autistics’ moral cognition with a well-validated moral judgment task.

### Utilitarian moral judgments on moral dilemmas

One widely used task that assesses the role of emotional processing in first-party, hypothetical harmful behaviours is the moral dilemma task^45^,^46^. Moral dilemmas are situations where two moral principles conflict with each other, e.g. “do not do harm unto others” against “act in a way so that maximum number of people will be better off”. In the harm domain, these dilemmas are instantiated by creating scenarios where the agent needs to act in order to produce the least harmful of possible outcomes (e.g., killing one to save many), i.e. situations where inaction would lead to more people getting hurt, but acting requires actively harming someone. These moral dilemmas are further divided into two classes based on the nature of harmful actions and their causal-intentional structure^47^ (see Table 1 for examples): (*i*) moral dilemmas that require agents to harm someone in up close and personal manner (by executing a motor act^48^) and where the victim needs to harmed as a *means* to achieve the greater good are called *personal* moral dilemmas (e.g., pushing someone to their death to save greater number of lives); while (*ii*) moral dilemmas that feature harms carried out not by physical force but by mechanical means and where the harm that befalls the victim is a *side-effect* of harmful act are called *impersonal* moral dilemmas (e.g., switching course of a trolley that kills someone to save more number of lives). Although the net outcome of choosing to act in both types of dilemmas can be the same (e.g., one life lost but five lives saved), most people endorse acting (which is said to be an utilitarian response) in cases of impersonal dilemmas but refuse to do so on personal dilemmas (which is said to be a deontological/non-utilitarian response^45^).

The dual-process model posits two types of processes that support each type of response in respective dilemma-contexts^45^: (*i*) automatic, affect-laden intuitions that surface as a reflex to aversive nature of the proposed harm and subserve non-utilitarian moral judgment; (*ii*) controlled, deliberative reasoning processes that engage in cost-benefit analysis and support utilitarian solution. Therefore, according to this model, individuals endorse utilitarian moral judgments more frequently on impersonal but not personal moral dilemmas because personal cases lead to a stronger negative affect in response to severe physical harm that needs to be carried out using personal force. There is plenty of evidence to support this claim^49^: neuroimaging^45^, psychophysiological^50^, and behavioural^51^ measures corroborate this model by revealing that indeed personal moral dilemmas elicit a more pronounced emotional response than the impersonal cases. Of interest to the current investigation, this negative emotional arousal partially stems from the harmful outcome, *viz.* empathic concern for the (to be sacrificed) victim’s pain which causes personal distress in the moral judge^25^.

Despite extensive use of this task in healthy controls, very little work has been carried out with the autistic population. Extensive prior work has focused on investigating moral cognition in clinical populations (e.g., patients with damage to the prefrontal cortex) and subclinical traits (e.g., psychopathy) characterized by social cognition and emotional processing disturbances using the moral dilemma task. These studies have consistently revealed that these populations have increased rate of utilitarian judgments on emotionally charged personal dilemmas as compared to control brain-damaged or neurotypical individuals^50^,^52^,^53^,^54^,^55^,^56^,^57^,^58^,^59^. Drawing on this prior work, one would expect that ASD would also beget a similar utilitarian moral profile due to similar sociocognitive and socioaffective problems.

Accordingly, one previous study has shown that ASD individuals are more willing to sacrifice someone for the greater good on personal moral dilemmas and report to perceive such situations to be less emotionally distressing as compared to controls, arguably due to reduced perspective-taking (cognitive empathy) that normally enables individuals to see things from the perspective of the person that needs to be sacrificed^11^. But this study used only one moral dilemma per condition and thus generalizability of these results remains to be assessed. This finding is also surprising in the light of evidence for prevalent negative hyperarousal in autistic individuals^60^,^61^,^62^, which would make it less likely that they would make utilitarian moral judgments^45^,^49^. Indeed, another unpublished study did not find any evidence for such increased utilitarian proclivity in ASD (Dr. Geoffrey Bird, personal correspondence).

The alexithymia hypothesis provides a plausible explanation for these conflicting findings in the extant work. Recent research shows that elevated level of subclinical alexithymia is associated with utilitarian profile on personal moral dilemma^41^, arguably due to reduced empathic concern for the victim that needs to be sacrificed^42^. Thus, it is possible that the prior finding about increased willingness to personally sacrifice someone for the greater good in ASD^11^ was due to presence of greater number of alexithymics in the ASD group as compared to healthy controls, since alexithymia is associated with both reduced perspective-taking and empathic concern for others^33^. Thus, increased tendency to endorse harmful sacrificial behaviours on moral dilemmas might have resulted from failure to empathize with the victim that needs to be sacrificed due to co-occurring alexithymia in ASD. Alternatively, it is also possible that utilitarian inclination due to alexithymic traits was counterbalanced by non-utilitarian inclination due to autistic traits. Severity of autism is associated with increased personal distress during demanding social situations^62^,^63^,^64^,^65^,^66^,^67^, which persists even after accounting for co-occurring alexithymia^64^, and this increased personal distress leads to withdrawal from engaging in personally carrying out harmful actions^68^,^69^. Thus, the nature of *between-group* differences in utilitarian moral judgment in a given study may depend on these *within-ASD-group* interactions between autistic and alexithymia traits that exert mutually opposite influence on utilitarian moral judgments.

Past work in autism also shows that autistics develop compensatory strategies from early childhood to counteract their lack of social intuitions^70^ whereby they strictly adhere to explicitly learned social rules and conventions in an inflexible or stereotyped manner^71^. This can also be garnered from overreliance on rule-based thinking while making distinction between (third-party) conventional and moral norm transgressions^9^,^10^, which are usually justified by healthy controls on the basis of considerations about victim suffering. Additionally, they rely less on emotional information and more on rule-based norm obedience while evaluating their own hypothetical choices about moral and prosocial behaviours^6^,^72^,^73^. Thus, it is possible that autistics rely on their intellectual abilities to form strategies that help them deal with complexities of distressing social environments and make adaptive decisions in such settings. This important aspect of their cognition has gone understudied in the past work and we explore its role in utilitarian moral judgments in the current study in concert with other personality traits.

### Predictions

Although we did not expect any group differences for utilitarian judgments on impersonal dilemmas based on prior work^11^, we did not have any *a priori* predictions regarding the between-group difference for utilitarian judgments on personal dilemmas in light of the conflicting findings from past studies. Indeed, in our framework, this difference can vary from study-to-study depending on the intricate web of mutually conflicting inputs from a composite of personality traits in the ASD sample (autism, alexithymia, intelligence measures, etc.).

We made following predictions for moral judgments in autistics on personal moral dilemmas: (*i*) alexithymic traits in the ASD sample would be associated with increased utilitarian inclination^41^,^42^ to endorse harmful sacrificial actions due to reduced empathic concern^33^,^34^,^35^,^39^,^74^,^75^,^76^,^77^,^78^,^79^; while (*ii*) autistic traits would be associated with reduced tendency to endorse utilitarian solution due to increased negative emotional arousal stemming from personal distress^68^,^69^ experienced by autistics while facing demanding social environments^62^,^63^,^64^,^65^,^66^,^67^. Note that although one may expect affective empathy (empathic concern and personal distress, i.e.) to predict *greater* endorsement for the utilitarian solution on personal dilemma due to greater empathizing with the many^78^- who would die in case of inaction - this is not observed because the utilitarian course of action features causal intervention on an identifiable and singular victim^80^ that needs to be sacrificed and thus the other set of victims are pushed to the background in the causal model and does not elicit a robust empathic response^81^,^82^. Additionally, we note that although autism is associated with increased personal distress even after accounting for co-occurring alexithymia^64^, trait alexithymia itself is also associated with greater personal distress but this association seems to be due to prevalent anxiety and is not characteristic of the alexithymic phenotype^33^.

Additionally, we expected there to be a negative correlation between intelligence measure and utilitarian moral judgments in ASD representing rigid rule-based norm abidance, but we were agnostic as to which component of IQ (verbal or non-verbal) would be implicated as a compensatory strategy and made this decision based on the exploratory correlation analysis.

Although recently a number of criticisms have surfaced that challenge interpreting affirmative response on moral dilemma as *utilitarian*^83^, we use utilitarian to mean “characteristically utilitarian” as a function of the response content and not the underlying motivation^49^. Thus, if a given individual responds affirmatively on a moral dilemma, we do not take this response to denote explicit endorsement of the utilitarian moral principle (“those acts are better that save more number of lives”) on her part, but only to mean that this response coincides with a response that would be endorsed by a typical, card-carrying utilitarian moral philosopher^49^.

## Methods

### Participants

The study sample consisted of 17 subjects (6 females) with a diagnosis of autism spectrum disorder (ASD group), who were recruited from autism-specific organizations, associations, and internet communities via various information materials (print flyers and posters, digital flyers, and Facebook advertisings) and had undergone a screening for any current comorbid psychiatric or medical condition. Importantly, we did not exclude ASD participants who were on medication - 7 subjects were consuming psychoactive drugs, primarily for depression. The medicated ASD group did not differ on any of the variables of interest from the non-medicated ASD group. The diagnosis was carried out by experienced clinicians according to the internationally accepted ICD-10 diagnostic criteria^84^. In line with a prior study^85^ and DSM-V^86^, we do not further divide ‘ASD group’ into ‘high-functioning autism’ and ‘Asperger’s Syndrome’ subgroups. We use the terms ‘autism’, ‘on the autism spectrum’, ‘autistic,’ and ‘autism spectrum disorder’ to refer to the ASD group as these terms are preferred by this population^87^.

Seventeen age-, gender- and level of education-matched participants (4 females; *χ*^2^(1) = 0.567, *p* = 0.452) were also included in the healthy controls (HC) group after an interview to ensure absence of history of drug abuse, neurological or other neuropsychiatric disorders. We note that although the final ASD group consisted of high-functioning autistic individuals with IQ comparable to the control group, the highest educational degrees that autistic individuals possessed tended to be slightly lower than the healthy controls (see Table 2).

All participants were financially compensated for their time and travel expenses and gave written informed consent. The study was approved by the local Ethics Committee (University of Vienna) and conducted in accordance with the declaration of Helsinki.

### Questionnaires

Various questionnaires (German-validated versions) were administered to assess individual differences in various aspects of the socioaffective processing: (*i*) Autism Spectrum Quotient (AQ) to assess severity of autistic traits^88^,^89^; (*ii*) Toronto Alexithymia Scale^90^,^91^ (TAS) to assess severity of alexithymic traits; (*iii*) Interpersonal Reactivity Index^26^,^92^ (IRI) as a self-report measure of trait empathy and Multifaceted Empathy Test^66^ (MET; revised version provided by I. Dziobek, personal correspondence) as a performance measure of state empathy; (*iv*) Emotion Regulation Questionnaire^93^,^94^ (ERQ) to assess emotion regulation profile; (*v*) Beck Depression Inventory^95^,^96^ (BDI) to assess severity of depression; (*vi*) short version of Raven’s Standard Progressive Matrices^97^,^98^ (SPM) and Mehrfachwahl-Wortschatz-Intelligenztest-B^99^,^100^ (MWT-B; Multiple Choice Vocabulary Intelligence Test) to assess non-verbal and verbal intelligence, respectively.

Good internal reliability was observed for subscales of questionnaires (see Table 2). For more detailed discussion about the questionnaires and their internal reliability analyses, see Supplementary Information (Text S1).

### Moral dilemma judgments

#### Stimuli

Experimental stimuli were text-based scenarios. There were three conditions representing each class of scenario: non-moral practical dilemmas (*n* = 6), impersonal moral dilemmas (*n* = 6), and personal moral dilemmas (*n* = 6) (see Table 1 for representative examples and Supplementary Information (Text S2) for detailed description of the scenarios). All scenarios featured first-person narrative.

Personal dilemmas featured situations that demanded agents (read participants) to carry out actions using personal force that violated others’ personal rights^48^. Compared to personal dilemmas, impersonal cases featured actions which were less emotionally salient and implicated the agent in the scenarios in less personal manner. The common denominator between moral dilemmas was that they pitted the normative injunction against violating someone’s individual rights by harming them in personal or impersonal manner against the utilitarian option of saving greater number of lives.

Non-moral scenarios posed practical questions and lacked any moral content. Data from non-moral scenarios are included in every model as a control condition. Thus, if any systematic differences are observed for moral dilemmas on any dependent variable, we can ascertain that this effect is specific to the moral domain by checking if the same effect is observed also for prudential, non-moral dilemmas.

#### Procedure

All participants were individually tested in a quiet room at the Faculty of Psychology of the University of Vienna. The experiment was carried out in two sessions separated on average by a week (*M*_ASD_ = 5.87 ± 3.02 days, *M*_HC_ = 6.13 ± 2.00 days, *t*(24.046) = −0.279, *p* = 0.783). In one session, participants completed the moral dilemma task; while in the other session, they completed another task (data not reported here). Similarly, in one session, participants completed AQ, IRI, TAS, and MET; while in the other session, participants completed ERQ and two other questionnaires (data not reported here). The moral tasks and questionnaire set pairings were randomized across sessions and participants. For the moral judgment task, before starting the actual experiment, each participant took part in one practice trial to ensure that they had understood all the instructions.

Moral judgment task and MET were administered on a computer, while the questionnaires were administered in paper-and-pencil format. The stimuli for the moral judgment tasks were presented using Cogent 2000 (Wellcome Department of Imaging Neuroscience, http://www.vislab.ucl.ac.uk/cogent.php) running on MATLAB platform. The text of the stories was presented in a black 21-point Arial font on a white background with a resolution of 800 × 600 pixels. MET task was presented using OpenSesame 2.8.1 program^101^ with a resolution of 1920 × 1080 pixels.

For the moral judgment task, the order of presentation of scenarios from each condition was randomized within subjects. Each dilemma description was presented in a single screen. Participants could read this screen at their own pace and move to the questions, by pressing the spacebar on the keyboard. The next two screens, presented in the same order for all participants, contained questions assessing: behavioural choice and emotional arousal (for exact wording, see Table 1). The behaviour and arousal questions lasted for as long as the participants needed. The affirmative answer on the behaviour question always corresponded to commission of sacrificial action. The spatial location (left or right arrows on the keyboard) of two options (yes or no) was constant across scenarios and subjects in order to avoid confusion and reduce working memory demands, especially for the ASD group. The emotional arousal ratings were recorded using a computerized visual analog scale (VAS), implemented as horizontal on-screen bar and responses were later converted to standardized scores with [min, max] of [0, 20].

We focused on behavioural choice of action (“Would you do it?”) over appropriateness of action (“Is it appropriate for you to do it?”) because: (*i*) it tends to be more emotionally arousing^102^, (*ii*) it tends to elicit more egocentric/self-focused (versus allocentric/other-focused) frame of reference because of potential self-relevant consequences^103^, and (*iii*) perceived appropriateness of utilitarian course of action on moral dilemmas does not differ in ASD^11^ (as compared to healthy controls). Thus, the behavioural choice of action provides a more sensitive measure to tap into moral cognition in autism.

Two ASD participants did not complete the moral dilemma task due to their unavailability for the second session, while data from one control participant could not be collected due to technical problems with MATLAB. The descriptive statistics (Table 2) thus include data only from these participants.

### Statistical analysis

All statistical analysis was carried out using JASP 0.7.1.12 (https://jasp-stats.org/). Effect size measures are reported as per prior recommendations^104^. All tests are two-tailed, unless otherwise stated. As recommended^105^, we provide univariate scatter-plots instead of bar graphs, especially given the small sample sizes in the current study. We follow recommended guidelines^106^ to ensure that our data met the statistical assumptions associated with the general linear model-based statistical tests.

Correlation analysis was carried out using Spearman’s *rho* as it is more robust to univariate outliers^107^ than Pearson’s *r*. To compare significance of within-group and between-group differences in correlations, we used Steiger’s and Fisher’s *Z*-tests respectively, as implemented in FZT-computator (http://psych.unl.edu/psycrs/statpage/FZT_backup.exe).

### Path analysis

In order to study complex web of interactions between different personality variables for utilitarian moral judgments, we conducted path analysis. Path analysis was performed in SPSS Amos 22 using maximum likelihood estimation^108^. Path analysis is a multivariate technique that requires formal specification of a model to be estimated and tested based on prior research and hypothesis. It involves specifying relationships between study variables and multiple equations denoting these relationships are solved simultaneously to test model fit and estimate parameters^108^. Note that path analysis is concerned only with *testing* the validity of theoretically-inspired models by fitting them to the observed data and not with *building* models^109^. As such, it cannot arbitrate as to whether the given model is correct or not, but only whether it fits the observed data. In the current study, path analysis was used to study divergent contributions of personality traits in utilitarian moral judgments in ASD. To this effect, models were constructed based on past work in the field and our theoretical predictions. The model fit was further improved by reducing model misspecification error with the inclusion of variables based on their correlation pattern with the variables of interest. As recommended^109^, model fit was not improved based on modification indices, but based on drawing paths that were theoretically meaningful.

All variables were standardized and centred before the analysis. Presence of multivariate outliers was investigated using Mahalanobis distance (none found). Since all paths represent linear relationships with a theoretically predicted direction, the significance threshold for regression coefficients associated with each path was determined based on one-tailed tests. Although there was a possibility of mediation effect involving some of the paths, no formal mediation analysis was carried out because the sample size was insufficient to carry out such analyses^110^.

In order to assess goodness of model fit, we chose indices that have been found to be least susceptible to effects of sample size, model misspecification, and parameter estimates. Following guidelines provided by Hooper and colleagues^111^, we used-(*i*) model chi-square and the root mean square error of approximation (RMSEA), along with the associated *p*-value for close fit, as the absolute fit indices (which measure the model fit in comparison to no model at all), (*ii*) comparative fit index (CFI) along with its parsimony index (PCFI) as the incremental fit indices (which gauge the model fit with respect to null model where all variables are uncorrelated). We do not report the standardized root mean square residual (SRMR) as Amos does not produce this index in the presence of missing data. The recommended cut-off values are^111^: RMSEA ≤ 0.07 (good), 0.07 < RMSEA ≤ 0.10 (moderate), *p* for close fit > 0.05, CFI ≥ 0.95. There is no recommended cut-off for PCFI.

## Results

### Elevated levels of alexithymia in ASD

As expected, ASD group had higher alexithymia score than the HC group (see Table 2). There were 7 autistics (out of 15 or 47%) who were also clinically alexithymic^90^ (≥54), while no participant from the control group scored above the clinical cut-off. The frequency of alexithymics differed significantly across groups (*χ*^2^(1) = 9.644, *p* = 0.002, *ϕ* = 0.558).

### Emotional processing deficits in ASD

As expected, ASD group was impaired (as compared to controls) on a number of emotional processing measures (see Table 2): (*i*) they reported to have reduced dispositional tendency to adopt others’ perspective and to experience increased personal distress in interpersonal interactions; (*ii*) they also exhibited maladaptive emotion regulation profile that relied more on suppressing emotion-expressive behaviour rather than reappraising emotional response; (*iii*) they did not exhibit any impairment on performance measures of empathy but did take longer to complete this task; (*iv*) they exhibited increased levels of depression.

Note that results from emotional processing measures are only briefly described here as data from these measures were ancillary to the main objective of the study. These results will be discussed in greater depth elsewhere.

### Moral dilemma task

The descriptive statistics for all variables associated with this task have been tabulated in Supplementary Information (Text S3). Although we had response time data, we do not draw any inferences about underlying psychological processes from analysis of this data as this practice of reverse inference has recently been demonstrated to be problematic^112^. Accordingly, analysis of response time data is provided in the Supplementary Information (Text S4). Suffice it to note here that there were no group differences for any condition and for any type of response (utilitarian or non-utilitarian).

### No group differences in behavioural choice on moral dilemmas

**Figure 1:**
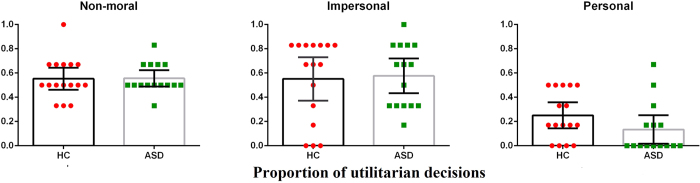
Summary of results for the behaviour question. Univariate scatter-plots (and corresponding bar-graphs) for proportion of affirmative responses on each type of scenario for each group for the behaviour question. For impersonal and personal moral dilemmas, higher scores indicate increased utilitarian tendency. Error bars represent 95% confidence intervals.

A 3 (condition: non-moral, impersonal, personal) × 2 (group) mixed ANOVA regarding behaviour question revealed a main effect of condition (*F*(1.536,44.534) = 31.736, *p* < 0.001, *pη*^2^ = 0.523, *ω*^2^ = 0.494), but there was neither a main effect of group (*F*(1,29) = 0.293, *p* = 0.593) nor a group-by-condition interaction effect (*F*(1.536,44.534) = 1.032, *p* = 0.347). Thus, autistics and controls did not differ in terms of their willingness to act in utilitarian manner on moral dilemmas. Of interest to us was personal moral dilemma on which autistics reported to be slightly less utilitarian than controls (see Fig. 1), although this difference was not significant (*t*(28.65) = 1.572, mean difference = −0.117, 95% CI [−0.268, 0.035], *p*(uncorrected) = 0.127, *d* = 0.566).

Decomposing the main effect of condition with planned Bonferroni-corrected comparisons revealed expected pattern of judgment for both groups: participants were more likely to be utilitarian on impersonal moral dilemmas as compared to personal moral dilemmas (HC: *t*(15) = 4.652, mean difference = 0.302, 95% CI [0.180,0.424], *p* < 0.001, *d* = 1.163; ASD: *t*(14) = 8.000, mean difference = 0.444, 95% CI [0.318,0.571], *p* < 0.001, *d* = 2.066) (see Fig. 1).

### Group differences in emotional arousal while facing moral dilemmas

A 3 (condition: non-moral, impersonal, personal) × 2 (group) mixed ANOVA for the arousal question revealed a main effect of condition (*F*(1.578,45.756) = 104.700, *p* < 0.001, *pη*^2^ = 0.783, *ω*^2^ = 0.771) but no condition-by-group interaction (*F*(1.578,45.756) = 0.250, *p* = 0.727). Planned comparisons revealed that both groups felt more emotionally aroused while facing scenarios from impersonal (HC: *t*(15) = 9.517, mean difference = 10.419, 95% CI [8.085,12.750], *p* < 0.001, *d* = 2.379; ASD: *t*(14) = 9.203, mean difference = 11.495, 95% CI [8.816,14.170], *p* < 0.001, *d* = 2.376) and personal (HC: *t*(15) = 7.096, mean difference = 8.476, 95% CI [5.930,11.020], *p* < 0.001, *d* = 1.774; ASD: *t*(14) = 6.161, mean difference = 9.336, 95% CI [6.086,12.590], *p* < 0.001, *d* = 1.591) dilemma conditions as compared to non-moral conditions. But both types of moral dilemmas were rated to be equally emotionally arousing (HC: mean difference = −1.942, *p* = 0.144; ASD: mean difference = −2.518, *p* = 0.096). Thus, autistics were not impaired in decoding emotional salience of different types of scenarios.

**Figure 2:**
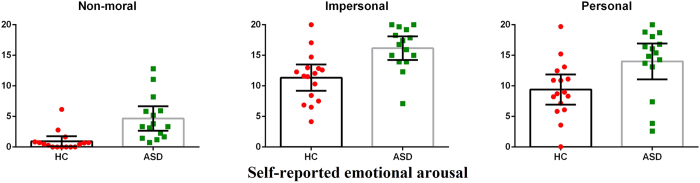
Summary of results for the emotional arousal question. Univariate scatter-plots (and corresponding bar-graphs) for self-reported emotional arousal (higher ratings denote more emotional arousal) while facing each type of scenario for each group. Error bars represent 95% confidence intervals.

Interestingly, there was also a main effect of group (*F*(1,29) = 16.720, *p* < 0.001, *pη*^2^ = 0.366, *ω*^2^ = 0.336). Bonferroni-corrected post-hoc comparisons revealed that ASD individuals found all scenarios to be more emotionally arousing than controls (non-moral: *t*(18.92) = 3.690, mean difference = 3.736, 95% CI [1.616,5.855], *p* = 0.006, *d* = 1.357; impersonal: *t*(28.81) = 3.552, mean difference = 4.812 , 95% CI [2.040,7.583], *p* = 0.003, *d* = 1.270; personal: *t*(27.88) = 2.556, mean difference = 4.596, 95% CI [0.912,8.279], *p* = 0.048, *d* = 0.923; see Fig. 2). Note that the emotional arousal was not specific to the moral domain, but was domain-general as would be expected based on prior studies^60^,^61^.

### Correlations analyses for utilitarian moral judgments on moral dilemmas

Correlations between moral judgments, arousal ratings, empathy, emotion regulation, personality traits, and intelligence measures were computed. Additionally, between-group differences in correlation patterns were investigated. Full details of these analyses are provided in Supplementary Information (Text S5–10).

**Figure 3:**
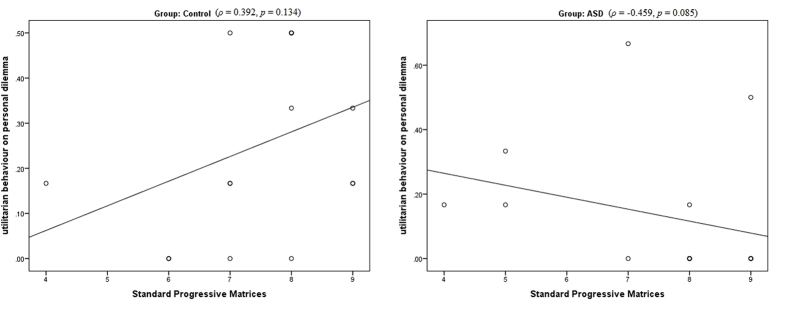
Non-verbal reasoning skills and moral judgments. The relation observed between non-verbal intelligence scores (as assessed by Raven’s Standard Progressive Matrices) and utilitarian moral judgment on personal moral dilemmas was diametrically opposite for the two groups (*Z* = 3.606, *p < *0.001). In controls, higher SPM scores were associated with a greater tendency to make utilitarian judgments, while autistics with higher SPM scores exhibited less favourable position for utilitarian option. Note that the number of data-points in the scatterplot seems to be less than the sample sizes due to overlap between data-points (denoted by circles with thicker circumference). Reported *p*-values are two-tailed.

In addition to the variables of a priori interest (AQ, TAS, EC, and PD), we used this correlation analyses to select additional variables that may have an influence on utilitarian moral judgments in ASD group. Interestingly, MWT-B was correlated negatively with utilitarian judgments on personal dilemmas in ASD (*ρ* = −0.739, *p* = 0.002), while SPM showed a marginally significant negative correlation (SPM: *ρ* = −0.459, *p* = 0.085). This pattern did not differ from the pattern observed in controls for MWT-B (*ρ* = −0.521, *p* = 0.039; *Z* = 0.926, *p* = 0.354), but it did differ for SPM (*ρ* = 0.392, *p* = 0.134; *Z* = 3.606, *p < *0.001). Thus, while higher general non-verbal intellectual abilities were associated with higher endorsement for utilitarian option on personal dilemmas in healthy controls, the pattern was exactly opposite in ASD participants such that higher SPM scores were predictive of reduced tendency to behave in utilitarian manner, although the correlation was only marginally significant (see Fig. 3; also see Supplementary Information (Text S11) for a similar scatterplot for MWT-B). No such group difference was observed for a measure of verbal intelligence. Thus, we selected SPM as a measure of non-verbal intelligence in our path model, since we suspected it was utilized by autistics as a compensatory strategy to cope with arousing social situations. We note that non-verbal IQ was chosen to represent a possible compensatory strategy not based on where it was significant or not, but based on the fact that the correlation between non-verbal IQ and moral judgment differed across groups.

### Path analysis of utilitarian moral judgments in ASD

In order to assess why utilitarian moral judgments were preserved on personal moral dilemmas in ASD despite the prevalent deficits in social cognition and emotional processing associated with this disorder, we formulated a path model for the different processes that were predicted to mediate mutually conflicting influences to leave the final moral judgment intact.

**Figure 4:**
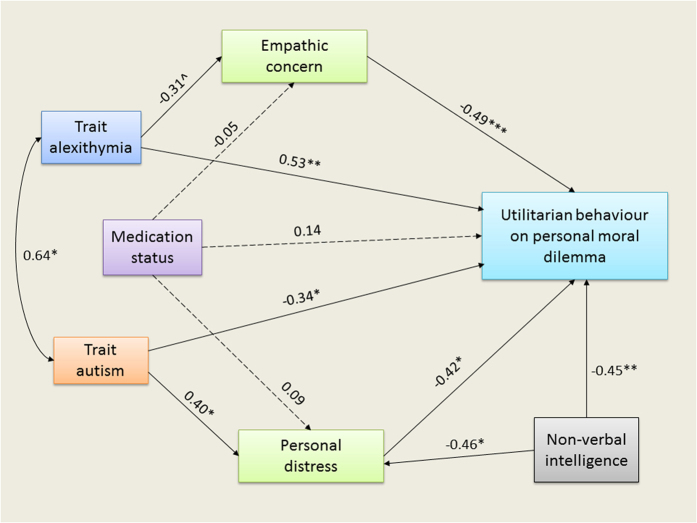
Path diagram from the path analysis model for utilitarian moral judgment. The path analysis model showing the divergent influences of autistic and alexithymic traits on utilitarian moral judgments on personal moral dilemmas in the ASD group, mediated by empathic concern and personal distress components of trait empathy. Additional variables accounted for effects of medication status (some autistics were consuming medication (=1), while some were not (=0)) and non-verbal reasoning scores (as assessed by Raven’s SPM).Values shown are standardized parameter estimates (betas). Although not shown in the figure, all endogenous variables are associated with errors. Solid lines represent significant relationships between predictors and the criterion variables, while dotted lines represent no significant relationship. Asterisks indicate significance of paths (^*p* < 0.1, *p < 0.05, **p < 0.01, ***p < 0.001, all one-tailed).

As mentioned before, alexithymic traits were predicted to be associated with increased utilitarian profile^41^,^42^ due to reduced empathic concern^33^,^34^,^39^,^42^, while autistic traits were expected to be associated with reduced utilitarian tendency on account of increased personal distress^62^,^63^,^64^,^65^,^66^,^68^,^69^ once shared variance between these two traits^28^,^29^,^30^,^31^,^32^ was controlled for. Additionally, we included SPM as a measure of intelligence since our correlation analyses showed that association between SPM and moral evaluation differed across groups and thus might index developmentally acquired, rule-based compensatory strategy to evaluate moral behaviour on hypothetical cases in ASD^6^,^10^,^72^,^73^. We also accounted for possible effects of medication^113^ status (dummy-coded as ON = 1, OFF = 0) on mediating variables; all effects of interest are observed even after exclusion of this variable and hence this variable was retained based on the improvement of the model fit. Although perspective-taking subscale of IRI has been implicated in increased utilitarian moral judgments on personal dilemmas in a prior ASD study^11^, we did not include it in the path analysis because-(*i*) none of the previous studies investigating predictive ability of different aspects of empathy (using IRI) in utilitarian moral judgments reveal any association between these two variables^42^,^68^,^75^,^76^,^77^,^78^,^79^, and (*ii*) inclusion of this variable led to a poor model fit (*p* < 0.05). Additionally, although we had both trait (IRI) and state (MET) measures of empathy we included only trait measures since a past study reveals that trait measures are better predictors of moral judgments on moral dilemmas than state measures^114^. Additionally, emotion regulation measures were not incorporated in the path model because they were not correlated with moral judgments in the current sample (Supplementary Information (Text S6)). The final model created with the inclusion of these variables is shown in Fig. 4.

This theoretically-inspired model exhibited a moderate fit to the data (*χ*^2^(9) = 10.007, *p* = 0.350, *χ*^2^/*df* = 1.112, RMSEA = 0.089, 90% CI [0,0.322], *p* for close fit = 0.378, CFI = 0.960, PCFI = 0.411). Together, the independent variables accounted for 69.5% of all variance (*R*^2^) in utilitarian moral judgments (for more details about betas from path analysis, see Supplementary Information (Text S12)).

As predicted, we found that once shared variance between autistic and alexithymic traits was accounted for, alexithymic traits exhibited increased affinity for personally carrying out the necessary harmful actions and autistic traits were associated with reduced tendency to endorse the utilitarian option. Furthermore, the influence of these two traits on moral judgments was mediated by dissociable components of empathy: (*a*) increased alexithymia score was associated with reduced dispositional empathic concern for others’ welfare (although this association was only marginally significant), which itself was associated with increased tendency to endorse utilitarian solution; (*b*) greater severity of autistic traits was associated with empathic hyperarousal in response to demanding social situation, which itself predicted reduced tendency to engage in harmful behaviour. Furthermore, greater capacity to reason non-verbally was also associated with reduced utilitarian behaviour.

Note that we did not carry out a similar path analysis with the control group because there was less amount of variation in personality traits (as compared to the ASD sample; see Table 2) to detect such subtle array of interactions between these traits (as assessed by Levene’s test, e.g., TAS: *F*(1,32) = 5.359, *p* = 0.027; personal distress: *F*(1,32) = 6.424, *p* = 0.016). Future studies should explore the same path model in a large control population with enough variation in the data to detect such interactions.

Since the estimates of the parameters are unstable in path analysis^109^ when the sample sizes are too small (like in the current study), we also assessed validity of the key results using a simpler model in a hierarchical regression analysis^6^ (for full details, see Supplementary Information (Text S13)). This analysis also revealed that after controlling for age, gender, and depression and after accounting for shared variance between autistic and alexithymic traits, severity of autism was associated with reduced utilitarian tendency (*β* = −0.701, *p* = 0.019), while alexithymia was predictive of increased utilitarian inclination (*β* = 0.840, *p* = 0.006).

## Discussion

Despite a large body of work investigating role of alexithymia in emotional processing deficits in autism^3^, its role in autistics’ moral cognition remains to be thoroughly explored. Moral cognition lies at the heart of interpersonal interactions and thus it in important to investigate this aspect of autistic cognition. In the current study, we explored moral evaluations in autistic participants on hypothetical, emotionally charged moral dilemmas that assessed their behavioural tendency to physically carry out harmful actions to avoid greater harm from occurring. Three primary results emerged from the current investigation. First, adults with ASD could properly distinguish between emotionally aversive personal dilemmas from impersonal dilemmas and endorsed behavioural choices that were comparable to controls. Second, autistic and alexithymia traits were associated with opposite utilitarian inclinations due to dissociable roles of self-oriented unease and other-oriented feelings of concern. Third, autistics relied on their intact non-verbal reasoning skills while making normative choices, probably to compensate for their other deficits in the interpersonal domain.

### Preserved utilitarian moral judgments in autism

As in healthy controls, ASD participants perceived making hypothetical choices on morally dilemmatic situations to be more emotionally arousing than finding solutions to practical problems and were more ready to endorse utilitarian option on impersonal as compared to personal moral dilemmas. Moreover, ASD participants found all conditions to be more arousing than controls, which comports well with prevalent negative arousal states reported in literature on autism^60^,^61^,^62^. Remarkably, this elevated negative emotional arousal and social and emotional processing deficits notwithstanding, not only did the autistic participants not show previously observed^11^ utilitarian bias, they exhibited increased tendency to *reject* the utilitarian option on emotionally salient dilemmas that required direct physical harm to a victim (e.g. pushing someone to their death). Our proposed framework premeditated such pattern of response based on a web of mutually conflicting influences of various subdimensions of autistic personality on first-hand, hypothetical moral choices.

### Dissociable empathy-utilitarianism associations between autistic and alexithymic traits

There is plenty of evidence to support the claim that emotions motivate individuals to reject harmful transgressions^23^,^115^, even if such actions are necessary to stave off harm of bigger magnitude^49^. Recent research also sheds light on the exact nature of psychological processes that constitute this negative affect^75^,^115^: aversion to harmful outcome (e.g. victim suffering) and aversion to the nature of harmful action itself (e.g. sensorimotor properties of the action). But the motivations subserving rejection of actions with harmful outcomes are of two varieties^68^,^76^: self-oriented personal distress and other-oriented empathic concern. Accordingly, since autistic traits are associated with increased personal distress^62^ during demanding interpersonal interactions (as shown by both self-reported ratings^65^,^66^,^67^ and hemodynamic responses^63^,^64^), we reasoned that their moral judgments would be influenced by this emotional bias *against* the utilitarian option^68^,^69^. On the other hand, since alexithymic traits are associated with reduced empathic concern for others’ wellbeing (as shown by both self-report^33^,^34^,^35^,^39^,^42^ and neuroimaging evidence^38^,^116^,^117^), they would be more likely to evaluate prospect of personally harming someone in a hypothetical scenario *in favour of* the utilitarian solution^42^,^76^. Thus, given the prevalence^28^,^29^,^30^,^31^,^32^ of alexithymia in ASD (in the current sample: 47%), we expected these dissociable empathic motivations mediating influences of autistic and alexithymia traits to cancel each other out leaving the final moral judgment unimpaired. This is exactly what was observed in the data, as shown by its fit to the theoretically-inspired path model (Fig. 4): egoistic motivation to reduce personal distress led to reduced utilitarian tendency for autistic traits, while reduced altruistic motivation to prevent harming others led to increased utilitarian proclivity for alexithymic traits. This model reveals that the spared moral capacity in autism to evaluate hypothetical harmful behaviours was a result of cancellation of opposite influences that are scaffolded on emotional biases introduced by dissociable empathic profiles of autistic and alexithymic traits. Thus, the current findings shed light not only on the different aspects of emotional empathy that autistic and alexithymic traits are associated with but also on how these traits relate to moral judgments.

We note that the current findings are in conflict with a prior study^6^ that investigated role of alexithymia in moral acceptability of emotion-evoking statements (e.g., “I could easily hurt you” (fear), “I never wash my hands” (disgust), etc.) and found that alexithymia was predictive of acceptability judgments only in controls but not in ASD and concluded that autistics’ judgments were based on complying with social rules and were less susceptible to emotional biases. It is possible that these differences stem from emotional saliency of the stimuli used across studies; moral dilemmas involve situations where the individuals have to mull over behavioural choice of directly harming or even killing someone for the benefit of the many and are, thus, inherently highly emotionally evocative^49^, while providing more objective acceptability judgments about emotional sentences may not engage emotional processes to the same extent^102^,^103^. Another possibility is that there was not enough variation in alexithymia scores in their ASD group to detect an effect (indeed, variance in alexithymia scores in the control group was higher than in the ASD group in the previous study^6^).

### Compensatory intellectual strategies in autism

Despite their social impairments, both children and adults with autism still manage to acquire knowledge about normative canon consisting of appropriateness of various moral behaviours^10^,^11^. For example, they can properly distinguish between moral norms that relate to suffering in victims from social conventions that are context-bound societal rules^7^,^8^,^9^,^10^. Although neurotypical individuals justify such distinction by referring to considerations about emotional consequences for the victim, the justifications provided by autistics tend to lack such empathic discourse and involve more rule-based rationale^9^,^10^,^72^,^73^. It is possible that in the absence of recourse to strong moral intuitions, autistics developmentally acquire compensatory strategies^70^ that rely on spared intellectual abilities; indeed research in moral development showing that children with intellectual disabilities lag behind their typically developing peers in terms of moral reasoning^118^ provides circumstantial evidence for this claim. Preserved intellectual ability can enable them to make such normatively significant distinctions by conforming to normative rules, sometimes in an inflexible and stereotyped manner^71^ which can make them adopt even harsher criterion for moral evaluations^10^,^12^. Accordingly, prior studies show that autistics exhibit a more rigid, rule-based profile to *justify* their moral choices on such tasks^9^,^10^ and enhanced verbal intelligence is predictive of quality of such justifications^119^,^120^, but these studies did not investigate role of such intellectual capabilities in moral *judgments*.

In the current study, we found that even after accounting for variance associated with autistic and alexithymic traits, non-verbal IQ was negatively predictive of utilitarian moral judgments. Thus, it is possible that autistics relied on non-verbal reasoning to reject the proposition of directly causing harm to others. For example, instead of retrieving semantic representations (for personal dilemma^45^, it can be “ME HURT YOU = WRONG”), they can rely on visual imagery of the same rule, which has indeed been shown to support non-utilitarian moral judgments in healthy individuals^121^. Prior studies support this line of reasoning, e.g., a previous neuroimaging study^122^ showed that typically developing children automatically encode their social knowledge into language while assessing behaviour of others in paradigms with minimum verbal requirements, but no such pattern is found in autistic children. Anecdotal reports from autistic individuals also note that they primarily rely on non-verbal thoughts^123^ (as one autistic noted^122^: “I think in pictures. Words are like a second language to me….When somebody speaks to me, his words are instantly translated into pictures”). The current findings are also consistent with the prior findings that show-(*i*) verbal IQ is correlated with *justifications* but not the moral *judgments* in children with ASD^119^,^120^, (*ii*) no correlation between verbal IQ and utilitarian moral judgments in ASD^11^, and (*iii*) some moral principles operative in moral evaluations seem to be inaccessible during conscious moral reasoning and seem to operate intuitively and are, thus, difficult to verbalize^124^.

Therefore, we maintain that the current findings hint at non-verbal intelligence as a compensatory strategy that high-functioning autistics rely on while endorsing moral choices that are in line with prevalent socio-moral norms. Although a prior study implicated intellectual abilities in forming compensatory strategies to perform a task in the perceptual domain^125^, no study thus far has investigated the same for the social domain and future hypothesis-driven studies should investigate the effect observed in the current study further.

### Implications

Current investigation underscores the importance of studying various aspects of cognition in clinical populations, even if they do not exhibit any visible deficits on the task being studied. More specifically, the current study raises a methodological concern for studies investigating moral cognition (especially in the harm domain) in clinical populations that have unusually high incidence rate of alexithymia^3^ (e.g., schizophrenia^126^, multiple sclerosis^40^, Parkinson’s disease^127^, etc.): all such studies should account for effects of co-occurring alexithymia on moral evaluations.

### Limitations

Validity of the conclusions drawn from the current study is contingent upon the following limitations. The primary limitation of the current study was the sample size, which was relatively small for the complexity of the statistical model investigated. Although we demonstrated validity of the main results in a separate regression analyses, future studies can explore various hypotheses stemming from the current investigation in a bigger sample (even in healthy population). Another limitation of the current study is the use of IRI to measure various components of empathy since the IRI items measuring empathic concern and personal distress do not seem to map well onto recent social neuroscience conception of empathy^25^ and also has psychometric problems^128^. Thus, the current findings should be replicated with other empathy measures. Additionally, the moral dilemma task has recently been criticized^83^ to have contexts that are too contrived and extreme to provide any cues about social behaviour in everyday life-like situations. We note though that such unfamiliar settings are especially helpful to shed light on processes that may not be robustly recruited while judging more mundane situations that can be resolved by easily accessible social rules^46^. Future studies can explore the role of alexithymia in reduced prosocial sentiments in autism using amore ecologically valid paradigm (e.g. ‘Above and Beyond’ task^72^,^73^), since this reduction in prosocial behaviour can be due to alexithymia^116^. Another limitation is that the current study used a single moral judgment parameter that treats utilitarian and deontological tendencies as inversely related to each other and conflate disregard for deontic prohibitions and endorsement of utilitarian principles and future studies should use process dissociation approach to study these separable appraisals^129^. Lastly, the diagnosis of autism was partially based on gold standard diagnostic instruments for ASD such as the Autism Diagnostic Interview – Revised^130^ (ADI-R) or the Autism Diagnostic Observation Schedule^131^ (ADOS) because these documents were not available for all participants and, therefore, an additional inclusion criterion was based on AQ-k. Future studies should attempt to include these standard diagnostic instruments as well.

## Additional Information

**How to cite this article**: Patil, I. *et al.* Divergent roles of autistic and alexithymic traits in utilitarian moral judgments in adults with autism. *Sci. Rep.* **6**, 23637; doi: 10.1038/srep23637 (2016).

## Supplementary Material

Supplementary Information

## Figures and Tables

**Table 1:** Three conditions from the moral dilemma task with representative examples from each category.

Condition	Non-moral	Impersonal	Personal
Text description	You have a very bad headache. You go to the pharmacy looking for your favorite brand of headache medicine. When you get there, you find that the pharmacy is out of the brand that you are looking for. You have known the pharmacist at this store for a long time, and you trust him. He says he has a generic medicine that is “exactly the same” as the name-brand medicine that you wanted. In the past, he has always given you good advice.	You are the driver of a runaway trolley approaching a fork in the tracks. On the tracks going to the left is a group of five railway workers. On the tracks going to the right is a single railway worker. If you do nothing, the trolley will go to the left, causing the five workers to die. The only way to avoid the deaths of these five workers is to hit a switch on your dashboard that will make the trolley go to the right, leading to the death of the single worker.	A runaway trolley is heading down the tracks toward five workers, and will kill them if it keeps going. You are on a footbridge over the tracks, in between the approaching trolley and the five workers. Next to you on this footbridge is a stranger who is very large. The only way to save the lives of the five workers is to push this stranger off the bridge and onto the tracks below where his large body will stop the trolley. The stranger will die if you do this, but the five workers will be saved.
Behaviour	Would you [nature of action] in order to [outcome of the proposed action]? (yes/no)		
Arousal	How emotionally arousing did you find this scenario? (0 = not at all arousing; 20 = extremely arousing)		

Each type of dilemma was followed by two questions: behaviour and emotional arousal. Impersonal and personal conditions involved moral content (implications for others’ wellbeing), while the non-moral cases involved only pragmatic issues.

**Table 2:** Descriptive statistic and group differences for various demographic, clinical, and experimental variables of interest (presented only for the participants included in the main analysis).

Variable	Cronbach’salpha	HC (*n* = 16)		ASD (*n* = 15)		Welch's *t*-test			
		Mean	SD	Mean	SD	*t*	*df*	*p*	Cohen’s *d*
*Clinical and demographic*									
Age	–	32.03	9.44	37.35	13.02	−1.295	25.43	0.207	−0.470
Education	–	4.50	1.41	3.40	1.92	1.807	25.67	0.083	0.656
SPM	–	7.44	1.32	7.53	1.64	−0.179	26.84	0.86	−0.065
MWT-B	–	29.94	2.82	31.13	4.21	−0.924	24.24	0.365	−0.336
BDI	–	3.25	2.35	9.53	7.81	−2.992	16.37	0.008	−1.106
*AQ-k*	0.954	5.69	3.00	24.87	3.44	−16.49	27.88	<0.001	−5.951
SIS	0.945	1.06	1.34	9.00	1.89	−13.41	25.10	<0.001	−4.873
IC	0.861	2.19	2.23	8.53	1.55	−9.25	26.85	<0.001	−3.286
CR	0.842	2.44	1.41	7.33	2.16	−7.42	23.89	<0.001	−2.701
*SPF-IRI*	0.658	50.31	6.10	50.80	8.32	−0.19	25.59	0.855	−0.067
Fantasy	0.683	13.00	2.68	10.87	3.40	1.93	26.65	0.064	0.700
Empathic Concern	0.748	13.94	3.23	13.40	3.02	0.48	29.00	0.636	0.172
Perspective-taking	0.756	14.38	2.68	11.73	2.91	2.62	28.36	0.014	0.945
Personal distress	0.804	9.00	1.93	14.80	3.55	−5.60	21.32	<.001	−2.049
*TAS*	0.863	34.75	3.96	53.60	8.63	−7.74	19.37	<0.001	−2.841
DIF	0.888	9.63	1.86	20.13	5.01	−7.64	17.56	<0.001	−2.817
DDF	0.844	11.38	2.19	20.20	2.51	−10.40	27.84	<0.001	−3.755
EOT	0.473	13.75	2.52	13.27	3.37	0.45	25.87	0.656	0.163
*ERQ*									
ERQ-Reappraisal	0.873	27.13	7.08	20.53	8.41	2.35	27.47	0.026	0.851
ERQ-Suppression	0.726	12.69	3.20	15.87	6.70	−1.67	19.78	0.111	−0.613
*MET*									
Cognitive-positive	–	16.50	3.18	15.53	1.68	1.066	23.09	0.298	0.376
Cognitive-positive-RT (in ms)	–	5563.28	1540.90	8609.84	3002.64	−3.519	20.59	0.002	−1.290
Cognitive-negative	–	14.38	2.39	15.07	3.39	−0.653	25.02	0.52	−0.237
Cognitive-negative-RT (in ms)	–	6103.65	2012.07	7979.87	2861.18	−2.099	24.98	0.046	−0.763
Affective-positive	–	5.56	1.55	4.11	1.44	2.691	29	0.012	0.965
Affective-positive-RT (in ms)	–	2933.40	1176.21	4663.17	2052.09	−2.855	22	0.009	−1.043
Affective-negative	–	5.47	1.02	4.82	1.86	1.207	21.36	0.241	0.442
Affective-negative-RT (in ms)	–	3796.17	1241.32	4819.58	2255.86	−1.551	21.46	0.136	−0.567

Notes: AQ-k – shortened version of Autism Spectrum Quotient; ASD - autism spectrum disorder; BDI - Beck Depression Inventory; CR - communication and reciprocity subscale of AQ-k; DDF - difficulty describing feelings; DIF - difficulty identifying feelings; EOT - externally-oriented thinking; ERQ - Emotion Regulation Questionnaire; HC - healthy controls; IC - imagination and creativity subscale of AQ-k; IRI - Interpersonal Reactivity Index; MET - Multifaceted Empathy Test; MWT-B - Mehrfachwahl-Wortschatz Intelligence Test; SPM - Raven’s Standard Progressive Matrices; RT - response time; SIS - social interaction and spontaneity subscale of AQ-k; SPF-IRI – German version of IRI; TAS - Toronto Alexithymia Scale total score.
