# Supplementary material for: Divergent roles of autistic and alexithymic traits in utilitarian moral judgments in adults with autism
Source: Sci Rep. 2016 Mar 29;6:23637. doi: 10.1038/srep23637 (PMC4810325; doi:10.1038/srep23637)
Supplement: Supplementary Information [file srep23637-s1.pdf]

Supplementary data for

"Divergent roles of autistic and alexithymic traits in utilitarian moral judgments in adults with autism"

Indrajeet Patil[1§*], Jens Melsbach[2§], Kristina Hennig-Fast[2], Giorgia Silani[1,2*]

[1]Scuola Internazionale Superiore di Studi Avanzati, Neuroscience Sector, Trieste, Italy.

[2]Department of Applied Psychology: Health, Development, Enhancement and Intervention, University of Vienna, Austria

[§]These authors contributed equally to this work.

*Correspondence should be addressed to: Indrajeet Patil, Neuroscience Sector, International School for Advanced Studies, SISSA-ISAS, Via Bonomea 265, 34136, Trieste, Italy. Email: patilindrajeet.science@gmail.com or

Giorgia Silani, Department of Applied Psychology: Health, Development, Enhancement and Intervention, Faculty of Psychology, University of Vienna, Liebiggasse 5, 1010 Vienna, Austria. Email: giorgia.silani@univie.ac.at

Abbreviations: AQ-k – shortened version of Autism Spectrum Quotient; AS – Asperger's Syndrome; ASD - autism spectrum disorder; BDI - Beck Depression Inventory; CI - confidence interval; CR - communication and reciprocity subscale of AQ-k; DDF - difficulty describing feelings; DIF - difficulty identifying feelings; EC - empathic concern; EOT - externally-oriented thinking; ERQ - Emotion Regulation Questionnaire; FS - fantasy; HC - healthy controls; HFA - High-functioning autism; IC - imagination and creativity subscale of AQ-k; IRI - Interpersonal Reactivity Index; MET - Multifaceted Empathy Test; MWT-B - Mehrfachwahl-Wortschatz Intelligence Test; PT - perspective taking; PD - personal distress; SPM - Raven's Standard Progressive Matrices; RT - response time; SIS - social interaction and spontaneity subscale of AQ-k; SPF-IRI – German version of IRI; TAS - Toronto Alexithymia Scale total score; ToM - Theory of Mind

Text S1: Questionnaires used and their internal reliability

Autism Spectrum Quotient (AQ-k): Autistic traits were assessed in all participants with the shortened, German-validated, 33-item version of the Autism Spectrum Quotient self-report questionnaire[1,2] designed for both clinical and community samples. This scale is further divided into three subscales: social interaction and spontaneity (SIS, 11 items; e.g. "I enjoy meeting new people"), imagination and creativity (IC, 12 items; e.g. "When I'm reading a story, I can easily imagine what the characters might look like"), communication and reciprocity (CR, 10 items; e.g. "I frequently find that I don't know how to keep a conversation going"). All questions were rated on a 4-point Likert scale from "Definitely Agree" to "Definitely Disagree" and were later recoded to 0 and 1.

Depression: Depressive symptoms in both groups were measured using Beck Depression Inventory[3,4].

Mehrfachwahl-Wortschatz-Intelligenz-Test (Multiple choice vocabulary test, *MWT*): MWT-B is the most commonly used version of MWT and is considered a measure of verbal intelligence[5,6]. It consists of 37 items and each item consists of five words (e.g. nesa - naso - nose - neso - nosa), out of which one authentic word needs to be recognized by the participants. Familiarity of the words varies widely and each correctly recognized word gives a point (thus possible scores range from 0 to 37). MWT-B has been shown to have good test-retest reliability and tends to be highly correlated with the other widely used measure of global IQ, viz. WAIS-Full-IQ.

Raven's Standard Progressive Matrices (SPM): SPM is considered a nonverbal estimate of fluid intelligence and the abbreviated version consisting of nine items (Form-A) was administered in the current study[7,8]. SPM items involve increasingly difficult pattern matching tasks and rely to a little degree on language abilities. Each correct answer is allotted one point and thus possible scores range from 0 to 9. This nine-item version has been shown to have good test-retest reliability and a high correlation with the full form SPM[8].

Toronto Alexithymia Scale (TAS): Interindividual differences in subclinical alexithymia were evaluated using the German-validated 18-item Toronto Alexithymia Scale[9,10] consisting of three subscales: Difficulty Identifying Feelings (DIF, 7 items; e.g., "When I am upset, I don't know if I am sad, frightened, or angry"), Difficulty Describing Feelings (DDF, 5 items; e.g., "It is difficult for me to find the right words for my feelings"), and Externally-Oriented Thinking (EOT, 6 items; e.g., "I prefer to analyze problems rather than just describe them"). Each item consisted of statements about emotional awareness and participants reported their agreement with these statements using a 5-point-Likert scale (1: *strongly disagree*, 5: *strongly agree*). TAS has been argued to be the best current measure overall for assessing alexithymia due to its sound reliability, validity, and broad generalizability[11].

Interpersonal Reactivity Index (IRI): The Interpersonal Reactivity Index[12,13] was used to assess specific aspects [fantasizing, empathic concern (EC), perspective-taking (PT), and personal distress (PD)] of dispositional empathy. The scale consisted of 16-items (four per subscale) and participants reported agreement with statements on a 5-point Likert scale (1: *never true for me*, 5: *always true for me*). Based on recent psychometric assessments of the IRI questionnaire[14], we *a priori* decided not to explore the fantasy subscale beyond descriptive statistics, as it does not map well onto the current neuroscientific discourse on empathy. Additionally, we focus on individual components of empathy rather than focusing on the entire construct of empathy since this approach provides more fine-grained understanding about empathy deficits in clinical populations.

Emotion Regulation Questionnaire (ERQ): Frequency of cognitive reappraisal and expressive suppression strategies to regulate emotions in everyday life was assessed with the Emotion Regulation Questionnaire[15,16]. Participants reported agreement with each statement using a 7-point Likert scale (1: *strongly disagree*, 7: *strongly agree*). Cognitive reappraisal is a cognitive strategy involving reinterpretation of events to reduce their emotional impact (6 items; e.g., ‘‘I control my emotions by changing the way I think about the situation I'm in.’’) and expressive suppression includes response-focused regulation involving inhibition of emotion-expressive behavior (4 items; e.g., ‘‘I control my emotions by not expressing them.’’).

Multifaceted Empathy Test (MET): To compliment the self-report paradigm (i.e., IRI), we also used a more naturalistic and ecologically valid performance measure, Multifaceted Empathy Test[17], to assess both cognitive and affective component of empathy for positive and negative emotions. We used the new, improved version MET-CORE-II (condensed and revised; Isabel Dziobek, personal correspondence) which includes 20 negative and 20 positive photographic stimuli (presented in blocks of either positive or negative emotional valence which consisted of 10 pictures randomized within each block) that depict people in emotionally charged contexts. In cognitive empathy condition (i.e., block 1, 4, 6, and 7: "What is this person feeling?"), participants had to choose an appropriate emotion from four available options (e.g., scared, despaired, confused, impatient.) and their accuracy and response times were recorded. In emotional empathy condition (i.e., block 2, 3, 5, and 8: "How much do you feel with this person?"), the degree of empathic concern participants felt for the person in the picture was assessed on a 9-point Likert scale (1: *not at all*, 9: *very much*) and response time data was also recorded.

Internal reliability:

Group differences in Cronbach's alphas were investigated using `cocron` package in R (http://comparingcronbachalphas.org/) which implements inferential statistics on alphas.

Scale	HC	ASD	Item coun	$\chi^2(1)$	*p*
AQ-k (*n* = 17)					
SIS	0.592	0.887	11	5.040	0.025
IC	0.635	0.753	12	0.507	0.477
CR	0.423	0.718	10	1.616	0.204
SPF-IRI (*n* = 17)					
FS	0.490	0.818	4	2.327	0.127
EC	0.765	0.564	4	0.863	0.353
PT	0.715	0.720	4	0.001	0.979
PD	0.545	0.741	4	0.720	0.396
TAS (*n* = 17)					
DDF	0.519	0.811	5	2.167	0.141
DIF	0.547	0.694	7	0.449	0.503
EOT	0.509	0.597	6	0.109	0.742
ERQ (*n* = 17)					
Reappraisal	0.865	0.819	6	0.239	0.625
Suppression	0.569	0.782	4	1.046	0.306
Moral dilemma task (*n* = 15)					
Impersonal -behavior	0.857	0.645	6	1.934	0.164
Personal - behavior	0.566	0.664	6	0.159	0.691

Text S2: Textual description of moral dilemmas

Impersonal and personal moral dilemmas were chosen from previously published batteries of moral dilemmas[18–21] and posed a conflict between actively harming less number of individuals for the welfare of many. In both impersonal and personal moral dilemmas, a number of factors that previous research has shown to affect moral judgments varied freely in order to increase heterogeneity and thus decrease predictability of experimental stimuli, e.g. whether the sacrificial actions benefited self or other[19], whether the victim's death was inevitable[21], kill-save ratios[22] (e.g., 1:6, 1:100s, etc.), etc.

Full descriptions of the scenarios used in the moral dilemma task are provided here. The German translations of the scenarios are available from the authors on request.

Non-moral scenarios

1. Two trips

You are bringing home some plants from the store. You have lined the trunk of your car with plastic to catch the mud from the plants, but your trunk will not hold all of the plants you have bought.

You could bring all of the plants home in one trip, but you would need to put some of the plants in the back seat. If you put the plants in the back seat, the mud from the plants will ruin your fine leather upholstery, which would cost thousands of dollars to replace.

2. Mutual Fund

You are at home one day when the mail arrives. You receive a letter from a company that provides financial services. You have heard of this company, which has a good reputation. They have invited you to invest in a mutual fund. The minimum investment for this fund is €1000.

You already know a lot about this particular mutual fund. It has performed poorly over the past few years. Based on what you know, there is no reason to think that it will perform any better in the future.

3. Jogging

You intend to accomplish two things this afternoon: going for a jog and doing some paperwork. In general you prefer to get your work done before you exercise.

The weather is nice at the moment, but the weather forecast says that in a couple of hours it will start to rain. You very much dislike jogging in the rain, but you don't care what the weather is like while you do paperwork.

4. Pharmacy

You have a very bad headache. You go to the pharmacy looking for your favorite brand of headache medicine. When you get there, you find that the pharmacy is out of the brand that you are looking for.

You have known the pharmacist at this store for a long time, and you trust him. He says he has a generic medicine that is "exactly the same" as the name-brand medicine that you wanted. In the past, he has always given you good advice.

5. Meeting

You need to travel to a nearby city in order to attend a meeting that starts at 2:00 PM. You can either take the train or the bus. The train will get you there just in time for your meeting no matter what.

The bus is scheduled to arrive an hour before your meeting. However, the bus sometimes is several hours late because of traffic. It would be nice to have an extra hour before the meeting, but it is very important that you arrive on time.

6. Scenery

An old friend invites you to spend the weekend at his summer home. This house is up the coast from where you live. You plan to drive, and you can take either the highway or the coastal road.

The highway will get you there in about three hours, but the scenery along the highway is very boring. The coastal road will get you to your friend's house in about three hours and fifteen minutes, and the scenery along the coastal road is very beautiful.

Moral impersonal

7. Hospital fumes

You are the late-night watchman in a hospital. Due to an accident in the building next door, there are deadly fumes rising up through the hospital's ventilation system. In a certain room of the hospital are three patients. In another room there is a single patient. If you do nothing the fumes will rise up into the room containing the three patients and cause their deaths.

The only way to avoid the deaths of these patients is to hit a certain switch, which will cause the fumes to bypass the room containing the three patients. As a result of doing this the fumes will enter the room containing the single patient, causing his death.

8. Trolley

You are the driver of a runaway trolley approaching a fork in the tracks. On the tracks going to the left is a group of five railway workers. On the tracks going to the right is a single railway worker.

If you do nothing, the trolley will go to the left, causing the five workers to die. The only way to avoid the deaths of these five workers is to hit a switch on your dashboard that will make the trolley go to the right, leading to the death of the single worker.

9. Motor boat

You are driving your motor boat in a small bay when your attention is drawn to cries of help from five people who are drowning at the end of a very narrow channel which is right in front of you. Between you and the people who are drowning, to one side of the channel, is another person who is calmly swimming.

If you do nothing, these five people would drown soon. You can steer towards the end of the channel at high speed. But you know that the person who is swimming in between will be hit by the motorboat and die, but the other five people will be saved.

10. Burning Car

You are the operator of a bridge in a huge parking complex. The bridge you operate connects two different floors. You see that an out of control car is approaching the bridge and is on fire. You see that on the top floor there are five people walking and on the bottom floor, there is one person walking. The floors they are walking on are so narrow that only one car can pass through at any given time. Right now, the bridge is in the upward position.

If you don't do anything, the car would continue its course on the top floor and kill those five people in its way. But if you move the bridge in the downward position, the car would go on the bottom floor and would kill the one person walking there.

11. Lifting magnet

You are the controller of a lifting magnet in a junkyard. Lifting magnets lift the heavy magnetic objects at some height and transport them to another place and drop them. The magnet, in automatic mode, is moving a car at some height from one place to another on the right side of the platform. Suddenly, you realize that if the magnet continues on its course, the magnet would drop the car on five people standing below. On the left, there is one person standing.

You can take control of the magnet. If you do nothing, the magnet would proceed to the right and drop the car attached to it, causing the death of the five people standing below. You can turn the magnet to left side of the platform, causing the death of the single person standing there.

12. Nurse

You are a nurse who is in charge of a machine which controls drug dosage levels in patients' blood. Because of a technical failure, the machine is supplying a lethal dose of the drug to four patients. Another patient, in a single room, is hooked up to the same machine and has not undergone any variation in dosage.

If nothing is done, these four patients would die due to lethal poisoning caused by drug overdose. You can press the button to block the drug supply to the four patients. You know that the overdose of drug will be redirected to the patient in the single room, who will die, but the other four will be saved.

Moral personal

13. Footbridge

A runaway trolley is heading down the tracks toward five workers, and will kill them if it keeps going. You are on a footbridge over the tracks, in between the approaching trolley and the five workers. Next to you on this footbridge is a stranger who is very large.

The only way to save the lives of the five workers is to push this stranger off the bridge and onto the tracks below where his large body will stop the trolley. The stranger will die if you do this, but the five workers will be saved.

14. Miners

You are leading a rescue team for seven miners that are stuck in an underground mine, which is flooding. Six miners are trapped at the bottom and will drown if not rescued soon. One miner is trapped higher in the elevator shaft and will not drown.

The only way to rescue the six at the bottom is to quickly send down the mine elevator. If you do this, the one miner in the shaft will be crushed to death. If you do not send down the elevator, you will have enough time to rescue the one miner in the shaft.

15. Vitamins

You are leading a group that is lost in the wilderness. Your group includes a family of six with a genetic vitamin deficiency. A few people’s kidneys contain large amounts of this vitamin. There is one such person in your group.

The only way to save the lives of the six family members is to remove one of this man’s kidneys and take the necessary vitamins from it. He will not die if you do this, but his health will get worse. He does not want to give his kidney, but you have the power to do what you choose.

16. Epidemic

An epidemic has spread worldwide killing millions of people. You have developed two substances in your underground shelter. One of them is a cure but the other one is deadly. You don’t know which is which.

Two people have run downstairs to your shelter trying to avoid the epidemic. The only way to identify the cure is to inject each of these people with one of the two substances. One person will live but the other will die. Then you will be able to start saving lives with the cure.

17. Savannah

You are carrying out research into the behaviour of lions in the Savannah of Central Africa. From an observation tower, you can see four people hiding behind a bush. They are being followed by a lion and are trying to get away from it. The lion has seen them and is heading for the bush. Meanwhile, another person has been able to climb the observation tower and is standing near you.

If you do nothing, the lion is going to find the four people hiding in the bush and kill them. You can push off the person standing near you so that the lion is drawn towards him. You know that this person will be mauled to death, but the other four will have time to escape.

18. Transplant

You are a young doctor working in the emergency surgery ward of your local hospital. Four of the patients under your care in your ward are dying from grave injury to vital organs as a result of a serious car accident. You have another patient in the ward who is now in good health and ready to be sent home.

If nothing is done, these four patients are going to die. You can anaesthetize the patient in good health and remove his vital organs to transplant them in the patients who are dying. You know that this patient will die, but the other four can be saved.

Text S3: Descriptive statistics for behavioral ratings for moral judgment tasks

Moral dilemma task									
**Question**	**Condition**	**HC (n = 16)**				**ASD (n = 15)**			
		**Mean**	**SD**	**Min**	**Max**	**Mean**	**SD**	**Min**	**Max**
behaviour	non-moral	0.55	0.17	0.33	1.00	0.56	0.12	0.33	0.83
	impersonal	0.55	0.34	0	0.83	0.58	0.26	0.17	1.00
	personal	0.25	0.20	0	0.50	0.13	0.21	0	0.67
emotional arousal	non-moral	0.93	1.58	0	6.16	4.66	3.61	0.72	12.80
	impersonal	11.35	4.05	4.16	20.00	16.16	3.49	7.09	20.00
	personal	9.40	4.64	0.05	19.71	14.00	5.32	2.61	20.00

Text S4: Details for response time data

Descriptive statistics and group differences for response time data from the moral dilemma task. No response time data was available for the arousal ratings. Note that although we had response time data for the moral dilemma task, we do not carry out any analysis on this data to make an inference about underlying psychological processes, as this practice of reverse inference has been recently demonstrated to be problematic[23].

scenario	Type of response	HC		ASD		*t*	*df*	*p*
		Mean	SD	Mean	SD			
non-moral	average	6.11	2.07	7.95	4.36	-1.485	19.71	0.153
impersonal	average	7.01	7.14	7.26	5.01	-0.114	26.94	0.910
personal	average	6.81	5.20	6.35	2.64	0.315	22.56	0.756
impersonal	utilitarian	4.23	3.19	8.20	6.89	-2.037	19.45	0.055
	non-utilitarian	6.90	7.72	7.39	4.81	-0.213	27.21	0.833
personal	utilitarian	5.55	2.12	6.68	2.76	-1.141	17.79	0.269
	non-utilitarian	5.70	3.02	7.91	4.00	-1.747	25.9	0.092
non-moral	utilitarian	11.05	13.13	5.78	1.58	1.378	11.38	0.195
	non-utilitarian	5.99	3.75	8.41	8.12	-1.051	19.43	0.306

Note: The reported *p*-values are uncorrected for multiple comparisons and would have to be adjusted for factorial design of the study.

Text S5: Correlation (Spearman's rho) between arousal ratings and moral judgments on moral dilemma task. *$p < 0.05$ (two-tailed)

Correlation pair	Control ($n = 16$)		ASD ($n = 15$)		Fisher's *Z*-test
	ρ	*p*	*ρ*	*p*	
Non-moral behaviour-arousal	-0.261	0.330	-0.397	0.142	0.382
Impersonal behaviour-arousal	0.219	0.414	**-0.583**	**0.023**	**2.222***
Personal behaviour-arousal	0.384	0.142	-0.150	0.594	1.389

Text S6: Correlation (Spearman's rho) between moral judgments on the moral dilemma task and arousal ratings and ERQ. *$p < 0.05$ (two-tailed)

Threshold of significance = 0.0167						Fisher's *Z*-test	
		HC (*n* = 16)		ASD (*n* = 15)			
variable	statistic	ERQ - reappraisal	ERQ - suppression	ERQ - reappraisal	ERQ - suppression	ERQ - reappraisal	ERQ - suppression
non-moral affirmative behaviour	*ρ*	.441	-.449	-.055	.214	1.32	1.751
	p	.087	.081	.845	.443		
impersonal utilitarian behaviour	*ρ*	.068	.454	.382	.100	0.835	0.973
	p	.802	.077	.160	.724		
personal utilitarian behaviour	*ρ*	.088	.278	.302	.450	0.558	0.498
	p	.745	.296	.274	.092		
non-moral emotional arousal	*ρ*	-.449	-.308	.070	-.250	1.383	0.157
	p	.081	.246	.805	.368		
impersonal emotional arousal	*ρ*	.386	-.320	.132	-.001	0.685	0.826
	p	.139	.228	.640	.997		
personal emotional arousal	*ρ*	.276	-.309	.317	.107	0.112	1.066
	p	.301	.244	.250	.703		

Text S7: Correlation (Spearman's rho) between moral judgments and arousal ratings and alexithymia. *$p < 0.05$ (two-tailed)

Threshold of significance = 0.0167				Fisher's *Z*-test
**variable**	**statistic**	**HC (n = 16)**	**ASD (n = 15)**	
non-moral affirmative behaviour	ρ	-.008	-.283	0.707
	p	.977	.307	
impersonal utilitarian behaviour	ρ	-.159	.030	0.476
	p	.556	.917	
personal utilitarian behaviour	ρ	-.088	.246	0.848
	p	.746	.378	
non-moral emotional arousal	ρ	.164	**0.765**	**2.105***
	p	.543	**.001**	
impersonal emotional arousal	ρ	-.181	.022	0.512
	p	.502	.937	
personal emotional arousal	ρ	-.059	-.165	0.268
	p	.827	.557	

Text S8: Correlation (Spearman's rho) between moral judgments and arousal ratings and SPF-IRI. *$p < 0.05$ (two-tailed)

Threshold of significance = 0.0167								Fisher's *Z*-test		
**variable**	**statistic**	**HC (n = 16)**			**ASD (n = 15)**					
		**EC**	**PT**	**PD**	**EC**	**PT**	**PD**	**EC**	**PT**	**PD**
non-moral affirmative behaviour	ρ	.483	.258	.382	-.377	-.062	-.014	**2.307***	0.814	1.04
	p	.058	.334	.144	.166	.827	.959			
impersonal utilitarian behaviour	ρ	-.173	.121	-.331	-.331	-.093	.212	0.423	0.537	1.397
	p	.522	.655	.210	.229	.742	.447			
personal utilitarian behaviour	ρ	.035	.212	-.193	-0.573	-.329	-.315	1.716	1.391	0.326
	p	.896	.431	.474	.026	.232	.253			
non-moral emotional arousal	ρ	-.313	-.388	-.088	-.018	-.018	.341	0.764	0.978	1.108
	p	.238	.138	.746	.949	.949	.213			
impersonal emotional arousal	ρ	.381	.322	.263	**0.641**	-.042	.227	0.896	0.939	0.096
	p	.146	.224	.325	**.010**	.881	.416			
personal emotional arousal	ρ	.151	.364	.070	0.523	-.178	.251	1.07	1.402	0.466
	p	.576	.166	.796	.045	.525	.367			

Text S9: Correlation (Spearman's rho) between moral judgments and arousal ratings and <u>MET performance</u>. *p < 0.05 (two-tailed)

		Threshold of significance = 0.0167								Fisher's *Z*-test			
		HC (n = 16)				ASD (n = 15)							
variable	statistic	Cognitive empathy positive correct answers	Cognitive empathy negative correct answers	Emotional empathy positive average	Emotional empathy negative average	Cognitive empathy positive correct answers	Cognitive empathy negative correct answers	Emotional empathy positive average	Emotional empathy negative average	Cognitive empathy positive correct answers	Cognitive empathy negative correct answers	Emotional empathy positive average	Emotional empathy negative average
non-moral affirmative behaviour	ρ	-.178	-.232	-.175	-.129	-.260	-.159	.270	-.008	0.215	0.19	1.133	0.304
	p	.509	.386	.516	.635	.350	.572	.330	.977				
impersonal utilitarian behaviour	ρ	.332	.381	-.275	0.000	-.152	-.272	.487	.225	1.245	1.699	**2.034***	0.572
	p	.209	.146	.303	1.000	.588	.326	.066	.421				
personal utilitarian behaviour	ρ	.361	.158	.040	-.015	-.025	-.147	.008	-.227	1.007	0.768	0.08	0.54
	p	.170	.560	.884	.957	.930	.600	.977	.417				
non-moral emotional arousal	ρ	-.286	-.309	0.573	-.195	.375	-.289	-.100	.150	1.72	0.055	1.879	0.871
	p	.283	.244	.020	.470	.168	.296	.723	.594				
impersonal emotional arousal	ρ	.261	.141	-.109	.237	.167	.423	-.148	.304	0.246	0.773	0.099	0.181
	p	.329	.603	.688	.376	.552	.116	.597	.271				
personal emotional arousal	ρ	-.028	-.012	.012	.071	-.226	.107	-.011	.236	0.505	0.298	0.057	0.423
	p	.918	.964	.966	.794	.418	.705	.970	.398				

Text S10: Correlation (Spearman's rho) between moral judgments and arousal ratings and personality traits. *$p < 0.05$ (two-tailed)

		**Threshold of significance = 0.0167**								**Fisher's *Z*-test**			
		**HC (*n* = 16)**				**ASD (*n* = 15)**							
**variable**	**statistic**	**AQ-*k***	**SPM**	**MWT-B**	**BDI**	**AQ-*k***	**SPM**	**MWT-B**	**BDI**	**AQ-*k***	**SPM**	**MWT-B**	**BDI**
non-moral affirmative	*ρ*	-.060	-.042	.102	-.418	-0.595	-.122	-.176	.129	1.562	0.201	0.7	1.436
behaviour	*p*	.825	.876	.707	.107	.019	.665	.531	.646				
impersonal utilitarian	*ρ*	-.358	**0.718**	-.047	.306	-.423	-.493	-.358	.041	0.192	**3.606***	0.818	0.687
behaviour	*p*	.173	**.002**	.862	.249	.116	.062	.190	.885				
personal utilitarian	*ρ*	-.281	.392	-0.521	.358	-.352	-.459	**-0.739**	-.096	0.197	**2.274***	0.926	1.176
behaviour	*p*	.293	.134	.039	.174	.198	.085	**.002**	.734				
non-moral emotional	*ρ*	.057	-.135	-.325	.364	**0.715**	-.006	-.059	.212	**2.099***	0.324	0.695	0.415
arousal	*p*	.833	.618	.219	.166	**.003**	.984	.834	.448				
impersonal emotional	*ρ*	.163	.406	-.368	-.046	.497	-.052	.408	.161	0.951	1.206	**2.047***	0.521
arousal	*p*	.546	.118	.160	.865	.060	.854	.131	.567				
personal emotional	*ρ*	-.111	.248	-.440	.058	.123	-.111	.292	.239	0.587	0.911	1.931	0.464
arousal	*p*	.682	.355	.088	.831	.663	.693	.291	.391				

Text S11: Correlation (Spearman's rho) between verbal reasoning skills and moral judgments

The relation observed between global/verbal intelligence scores (as assessed by MWT-B) and utilitarian moral judgment on personal moral dilemmas was similar for the two groups (Z = 0.926, p = 0.354). In both controls and ASD group, higher MWT-B scores were associated with a reduced tendency to make utilitarian judgments. Note that the number of data-points in the scatterplot seems to be less than the sample sizes due to overlap between data-points (denoted by circles with thicker circumference). Reported p-values are two-tailed.

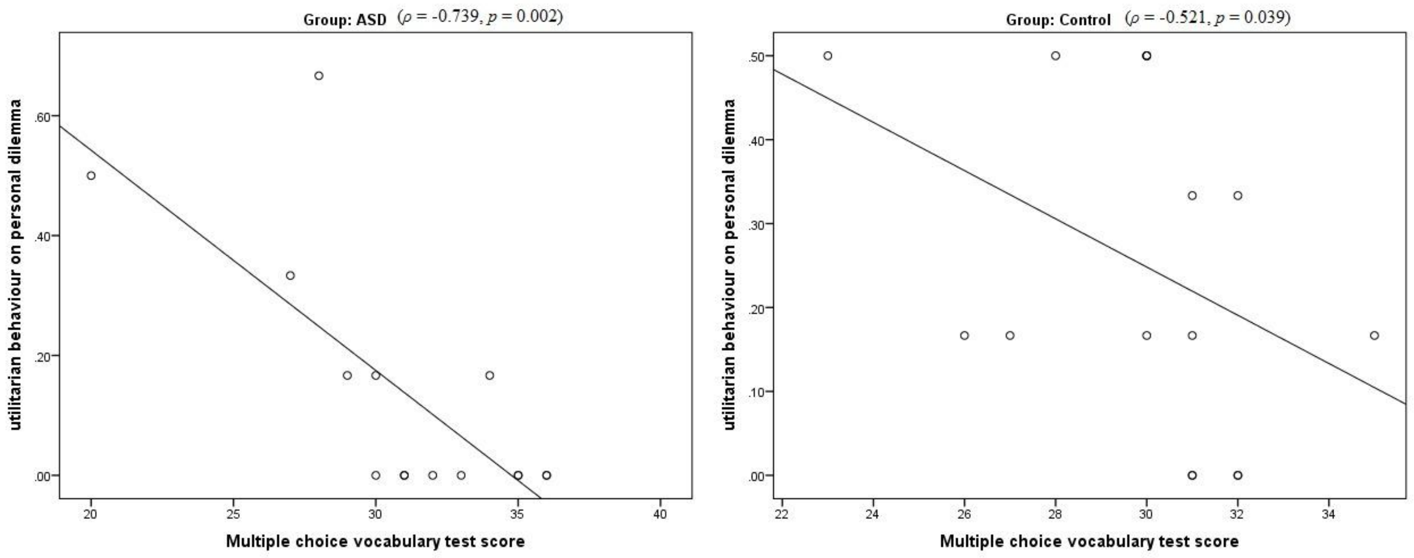

Text S12: Details for the standardized regression coefficients for each path from the path analysis model

Since we had directional hypotheses for most paths, one-tailed p-values have been provided. *Note*: S.E. – Standard Error, MJ – moral judgment for the behavior question on the personal moral dilemma

			β	p (1-tailed)	S.E.
EC	<---	TAS	-0.313	0.100	0.254
PD	<---	AQ	0.396	0.030	0.214
PD	<---	SPM	-0.461	0.015	0.214
PD	<---	Medication status	0.091	0.334	0.422
EC	<---	Medication status	-0.047	0.426	0.501
MJ	<---	AQ	-0.338	0.049	0.175
MJ	<---	TAS	0.535	0.004	0.168
MJ	<---	EC	-0.491	0.001	0.132
MJ	<---	PD	-0.418	0.013	0.157
MJ	<---	SPM	-0.452	0.004	0.146
MJ	<---	Medication status	0.142	0.171	0.250

Text S13

Hierarchical regression analyses (conducted separately in the control and ASD groups) determined whether alexithymia or ASD symptom severity predicted utilitarian responses on the behavior question for personal moral dilemmas once age, gender, and depression were controlled for, and whether each could predict the dependent variables after the other was controlled for. As noted in a previous study[24], it is necessary to perform hierarchical regressions with alexithymia and ASD symptom severity entered in both possible orders to independently investigate the effect of each, after controlling for the other, because of collinearity. We acknowledge that limitation of the following analysis is again that the sample size is smaller than recommended[25]. Note: All reported *p*-values are from two-tailed tests.

- <u>For ASD group (*n* = 15) with order ASD severity first, alexithymia second</u>

**Step**	**Predictor**	**ASD group : behavior ratings for personal dilemma**				
		β	t	p	R^2	ΔR^2 (p)
1	(Constant)		2.704	0.021	31.2%	31.2% (0.232)
	Gender	-0.193	-0.751	0.468		
	Age	-0.498	-1.915	0.082		
	Depression	-0.186	-0.725	0.483		
2	(Constant)		1.621	0.136	32.6%	1.4% (0.657)
	Gender	-0.164	-0.599	0.563		
	Age	-0.472	-1.712	0.118		
	Depression	-0.166	-0.614	0.553		
	ASD severity	-0.126	-0.457	0.657		
3	(Constant)		1.120	0.292	72.2%	39.6% (0.006)
	Gender	-0.170	-0.919	0.382		
	Age	-0.319	-1.664	0.130		
	Depression	-0.073	-0.394	0.703		
	ASD severity	-0.701	-2.846	0.019		
	Alexithymia	0.840	3.583	0.006		

- For ASD group (n = 15) with order alexithymia first, ASD severity second

Step	Predictor	ASD group : behavior ratings for personal dilemma				
		β	t	p	R^2	ΔR^2 (p)
1	(Constant)		2.704	0.021	31.2%	31.2% (0.232)
	Gender	-0.193	-0.751	0.468		
	Age	-0.498	-1.915	0.082		
	Depression	-0.186	-0.725	0.483		
2	(Constant)		-0.080	0.938	47.2%	16% (0.112)
	Gender	-0.259	-1.087	0.303		
	Age	-0.481	-2.011	0.072		
	Depression	-0.186	-0.788	0.449		
	Alexithymia	0.406	1.743	0.112		
3	(Constant)		1.120	0.292	72.2%	25% (0.019)
	Gender	-0.170	-0.919	0.382		
	Age	-0.319	-1.664	0.130		
	Depression	-0.073	-0.394	0.703		
	Alexithymia	0.840	3.583	0.006		
	ASD severity	-0.701	-2.846	0.019		

As can be noted from the regression coefficients, both autistic and alexithymic traits were significant predictors of the utilitarian moral behavior on personal dilemmas, but with opposite signs. That is, greater severity of autistic traits was associated with increased endorsement of the option of inaction, while higher scores on alexithymia were characterized by greater endorsement of the utilitarian choice. Thus, the pattern revealed by complex path analysis was also observed in this simpler regression analysis.

Next, we investigated if the same pattern was also observed in the control sample. As mentioned in the main text, there was less amount of variation in trait alexithymia in control sample as compared to ASD sample (SD_{ASD} = 8.091, SD_{HC} = 3.890; Levene's test: $F(1,32) = 5.359$, $p = 0.027$), but not for AQ-k scores

(SD_{ASD} = 3.238, SD_{HC} = 3.182; Levene's test: $F(1,32) = 0.046$, $p = 0.831$). This was a result of our methodological choice to not match the two groups for alexithymia scores (in contrast to prior recommendations[26]), since this requires oversampling the control group and consequently is unrepresentative with respect to distribution of alexithymic traits in this population. Given that there was not enough variation in TAS scores in control group with respect to the ASD group, but equivalent variation in AQ scores, we expected analogous result in the HC group only for the AQ but not TAS traits. We did not carry out moderation analysis to see if factor group moderated relationship between alexithymia severity and utilitarian judgments (like in a previous study[24]), because the two groups were not matched for alexithymia scores and the current study also lacked power to detect such effect.

- For HC group (n = 16) with order ASD severity first, alexithymia second

Step	Predictor	HCgroup : behavior ratings for personal dilemma				
		β	t	p	R^2	ΔR^2 (p)
1	(Constant)		5.369	< 0.001	69.4%	69.4% (0.002)
	Gender	-0.217	-1.285	0.223		
	Age	-0.788	-4.728	< 0.001		
	Depression	0.234	1.422	0.180		
2	(Constant)		8.511	< 0.001	86.3%	16.9% (0.004)
	Gender	-0.299	-2.493	0.030		
	Age	-0.848	-7.216	< 0.001		
	Depression	0.254	2.204	0.050		
	ASD severity	-0.423	-3.693	0.004		
3	(Constant)		4.405	0.001	86.4%	0.1% (0.890)
	Gender	-0.300	-2.383	0.038		
	Age	-0.844	-6.709	< 0.001		
	Depression	0.254	2.108	0.061		
	ASD severity	-0.418	-3.378	0.007		
	Alexithymia	-0.017	-0.142	0.890		

As can be seen from the final model, gender emerged as a significant negative predictor of utilitarian judgment, i.e. women were less likely to judge harming the few for the greater good than men, which agrees with a recent large-scale meta-analysis[27]. Additionally, it was also observed that older people were less likely to endorse utilitarian judgment, but this result might be an artifact of small sample size as prior surveys with study sample large enough to investigate age-related variation on moral judgments revealed null results[28].

- For HC group (*n* = 16) with order alexithymia first, ASD severity second

**Step**	**Predictor**	**HC group : behavior ratings for personal dilemma**				
		β	t	p	R^2	ΔR^2 (p)
1	(Constant)		5.369	< 0.001	69.4%	69.4% (0.002)
	Gender	-0.217	-1.285	0.223		
	Age	-0.788	-4.728	< 0.001		
	Depression	0.234	1.422	0.180		
2	(Constant)		3.060	0.011	70.8%	1.4% (0.481)
	Gender	-0.227	-1.313	0.216		
	Age	-0.767	-4.444	0.001		
	Depression	0.241	1.434	0.179		
	Alexithymia	-0.122	-0.729	0.481		
3	(Constant)		4.405	0.001	86.4%	15.6% (0.007)
	Gender	-0.300	-2.383	0.038		
	Age	-0.844	-6.709	< 0.001		
	Depression	0.254	2.108	0.061		
	Alexithymia	-0.017	-0.142	0.890		
	ASD severity	-0.418	-3.378	0.007		

As expected, we found evidence for decreased non-utilitarian tendency with autistic traits also in the control sample, but no evidence for alexithymic traits due to lack of enough variation in these traits. A prior study done with healthy sample did reveal utilitarian bias in trait alexithymia[29], but this study did not investigate the role of autistic traits. Future studies should investigate divergent contributions of these two traits in a large sample consisting of healthy adults.

References

1. Baron-Cohen, S., Wheelwright, S., Skinner, R., Martin, J. & Clubley, E. The Autism-Spectrum Quotient (AQ): Evidence from Asperger Syndrome/High-Functioning Autism, Males and Females, Scientists and Mathematicians. *J. Autism Dev. Disord.* **31,** 5–17 (2001).

2. Freitag, C. M. *et al.* German adaptation of the autism-spectrum quotient (AQ): Evaluation and short version AQ-k. *Zeitschrift fur Klin. Psychol. und Psychother. Forsch. und Prax.* **36,** 280–289 (2007).

3. Beck, A. T., Steer, R. A. & Brown, G. K. Manual for the Beck depression inventory-II. *San Antonio, TX Psychol. Corp.* 1–82 (1996).

4. Hautzinger, M. Das Beck-Depressions-Inventar (BDI) in der Klinik. *Nervenarzt* **62,** 689–96 (1991).

5. Lehrl, S., Triebig, G. & Fischer, B. Multiple choice vocabulary test MWT as a valid and short test to estimate premorbid intelligence. *Acta Neurol. Scand.* **91,** 335–345 (1995).

6. Lehrl, S. *Mehrfachwahl-Wortschatz-Intelligenztest: MWT-B.* (PERIMED-spitta, 1995).

7. Raven, J., Raven, J. C. & Court, J. *Manual for Raven's progressive matrices and vocabulary scales*. (1998).

8. Bilker, W. B. *et al.* Development of abbreviated nine-item forms of the Raven's standard progressive matrices test. *Assessment* **19,** 354–69 (2012).

9. Bagby, R. M., Parker, J. D. & Taylor, G. J. The twenty-item Toronto Alexithymia Scale--I. Item selection and cross-validation of the factor structure. *J. Psychosom. Res.* **38,** 23–32 (1994).

10. Kupfer, J., Brosig, B. & Brähler, E. Überprüfung und Validierung der 26-Item Toronto Alexithymie-Skala anhand einer repräsentativen

Bevölkerungsstischprobe. *Z. Psychosom. Med. Psychother.* **46,** 368–384 (2000).

11. Timoney, L. R. & Holder, M. D. *Emotional Processing Deficits and Happiness: Assessing the Measurement, Correlates, and Well-Being of People with Alexithymia*. *Emotional Processing Deficits and Happiness* (2013). doi:10.1007/978-94-007-7177-2

12. Davis, M. H. Measuring individual differences in empathy: Evidence for a multidimensional approach. *J. Pers. Soc. Psychol.* **44,** 113–126 (1983).

13. Paulus, C. Der Saarbrücker Persönlichkeitsfragebogen SPF (IRI) zur Messung von Empathie: Psychometrische Evaluation der deutschen Version des Interpersonal Reactivity. (2009).

14. Baldner, C. & McGinley, J. J. Correlational and exploratory factor analyses (EFA) of commonly used empathy questionnaires: New insights. *Motiv. Emot.* **38,** 727–744 (2014).

15. Gross, J. J. & John, O. P. Individual differences in two emotion regulation processes: implications for affect, relationships, and well-being. *J. Pers. Soc. Psychol.* **85,** 348–362 (2003).

16. Abler, B. & Kessler, H. Emotion Regulation Questionnaire - Eine Deutschsprachige Fassung des ERQ von Gross und John. *Diagnostica* **55,** 144–152 (2009).

17. Dziobek, I. *et al.* Dissociation of cognitive and emotional empathy in adults with Asperger syndrome using the Multifaceted Empathy Test (MET). *J. Autism Dev. Disord.* **38,** 464–473 (2008).

18. Greene, J., Nystrom, L. E., Engell, A. D., Darley, J. M. & Cohen, J. D. The neural bases of cognitive conflict and control in moral judgment.

Neuron **44,** 389–400 (2004).

19. Lotto, L., Manfrinati, A. & Sarlo, M. A New Set of Moral Dilemmas: Norms for Moral Acceptability, Decision Times, and Emotional Salience. *J. Behav. Decis. Mak.* **27,** 57–65 (2014).

20. Patil, I., Cogoni, C., Zangrando, N., Chittaro, L. & Silani, G. Affective basis of judgment-behavior discrepancy in virtual experiences of moral dilemmas. *Soc. Neurosci.* **9,** 94–107 (2014).

21. Moore, A. B., Clark, B. A. & Kane, M. J. Who shalt not kill? Individual differences in working memory capacity, executive control, and moral judgment. *Psychol. Sci.* **19,** 549–557 (2008).

22. Trémolière, B. & Bonnefon, J.-F. Efficient Kill-Save Ratios Ease Up the Cognitive Demands on Counterintuitive Moral Utilitarianism. *Pers. Soc. Psychol. Bull.* **40,** 923–930 (2014).

23. Krajbich, I., Bartling, B., Hare, T. & Fehr, E. Rethinking fast and slow based on a critique of reaction-time reverse inference. *Nat. Commun.* **6,** 7455 (2015).

24. Brewer, R. *et al.* The Impact of Autism Spectrum Disorder and Alexithymia on Judgments of Moral Acceptability. **124,** 589–595 (2015).

25. Maxwell, S. E. Sample size and multiple regression analysis. *Psychol. Methods* **5,** 434–58 (2000).

26. Bird, G. & Cook, R. Mixed emotions: the contribution of alexithymia to the emotional symptoms of autism. *Transl. Psychiatry* **3,** e285 (2013).

27. Friesdorf, R., Conway, P. & Gawronski, B. Gender Differences in Responses to Moral Dilemmas: A Process Dissociation Analysis. *Personal. Soc.*

Psychol. Bull. **41,** 696–713 (2015).

28. Hauser, M., Cushman, F., Young, L., Jin, R. K.-X. & Mikhail, J. A Dissociation Between Moral Judgments and Justifications. *Mind Lang.* **22,** 1–21 (2007).

29. Patil, I. & Silani, G. Alexithymia increases moral acceptability of accidental harms. *J. Cogn. Psychol.* **26,** 597–614 (2014).
